# Comparative efficacy of early TIPS, Non-early TIPS, and Standard treatment in patients with cirrhosis and acute variceal bleeding: a network meta-analysis

**DOI:** 10.1097/JS9.0000000000000865

**Published:** 2023-11-03

**Authors:** Ye Huang, Xiaokai Wang, Xiangmin Li, Shichang Sun, Yongxiang Xie, Xinbo Yin

**Affiliations:** a Teaching and Research Section of Clinical Nursing, Xiangya Hospital, Central South University; b National Clinical Research Center for Geriatric Disorders, Xiangya Hospital, Changsha, Hunan, P.R. China; c Department of Emergency Medicine, Xiangya Hospital, Central South University, Changsha, China; d Loudi Center for Diseases Prevention and Control, Loudi, Hunan, China

**Keywords:** acute variceal bleeding, cirrhosis, early transjugular intrahepatic portosystemic shunt, network meta-analysis, standard treatment

## Abstract

**Background:**

Cirrhosis is a chronic disease characterized by chronic liver inflammation and diffuse fibrosis. A combination of vasoactive drugs, preventive antibiotics, and endoscopy is the recommended standard treatment for patients with acute variceal bleeding; however, this has been challenged. We compared the effects of early transjugular intrahepatic portosystemic shunt (TIPS), non-early TIPS, and standard treatment in patients with cirrhosis and acute variceal bleeding.

**Materials and Methods:**

The present network meta-analysis was conducted in accordance with the criteria outlined in the Preferred Reporting Items for Systematic Reviews and Meta-Analyses and Assessing the methodological quality of systematic reviews guidelines. The review has been registered with the International Prospective Register of Systematic Reviews. The PubMed, Embase, Cochrane Library, ClinicalTrials.gov, and World Health Organization-approved trial registry databases were searched for randomized controlled trials (RCTs) evaluating early TIPS, non-early TIPS, and standard treatment in patients with cirrhosis and acute variceal bleeding.

**Results:**

Twenty-four RCTs (1894 patients) were included in the review. Compared with standard treatment, early TIPS [odds ratio (OR), 0.53; 95% credible interval (Cr), 0.30–0.94; surface under the cumulative ranking curve (SUCRA), 98.3] had a lower risk of all-cause mortality (moderate-to-high-quality evidence), and early TIPS (OR, 0.19; 95% CrI, 0.11–0.28; SUCRA, 98.2) and non-early TIPS (OR, 0.30; 95% CrI, 0.23–0.42; SUCRA, 1.8) were associated with a lower risk of rebleeding (moderate-to-high-quality evidence). Early TIPS was not associated with a reduced risk of hepatic encephalopathy, and non-early TIPS (OR, 2.78; 95% CrI, 1.89–4.23, SUCRA, 0) was associated with an increased incidence of hepatic encephalopathy (moderate-to-high-quality evidence). There was no difference in the incidence of new or worsening ascites (moderate-to-high-quality evidence) among the three interventions.

**Conclusion:**

Based on the moderate-to-high quality evidence presented in this study, early TIPS placement was associated with reduced all-cause mortality [with a median follow-up of 1.9 years (25th–75th percentile range 1.9–2.3 years)] and rebleeding compared to standard treatment and non-early TIPS. Although early TIPS and standard treatment had a comparable incidence of hepatic encephalopathy, early TIPS showed superiority over non-early TIPS in this aspect. Recent studies have also shown promising results in controlling TIPS-related hepatic encephalopathy. However, it is important to consider individual patient characteristics and weigh the potential benefits against the risks associated with early TIPS. Therefore, we recommend that clinicians carefully evaluate the patient’s condition, considering factors such as severity of variceal bleeding, underlying liver disease, and overall clinical status, before making a treatment decision. Further well-designed RCTs comparing early TIPS with non-early TIPS are needed to validate these findings and provide more definitive guidance.

## Introduction

Highlights

Early transjugular intrahepatic portosystemic shunt (TIPS) has a better therapeutic effect in patients with cirrhosis and acute variceal bleeding.
Early TIPS is associated with a lower risk of rebleeding and mortality than standard treatment or non-early TIPS.
Early TIPS is associated with a lower risk of hepatic encephalopathy than non-early TIPS.

Cirrhosis is a chronic disease characterized by chronic liver inflammation and diffuse fibrosis. Consequently, the normal liver structure is replaced by regenerative liver nodules, leading to portal hypertension^1^. Gastrointestinal bleeding is a serious complication of portal hypertension and is the most common cause of bleeding; it is also the main cause of death in patients with liver cirrhosis^2^. The prevalence of esophageal varices in patients with liver cirrhosis is 50–60%, increasing to as high as 85% in patients with decompensated cirrhosis, and the risk of death is 15–30% within 6 weeks, especially higher when infection is present^3,4^. Therefore, early control of variceal bleeding is essential to reduce mortality in patients with non-transplant cirrhosis^5^. At present, a combination of vasoactive drugs, preventive antibiotics, and endoscopic techniques is the recommended standard treatment for patients with acute variceal bleeding^6,7^.

However, the standard treatment has been challenged because transjugular intrahepatic portosystemic shunt (TIPS) has been demonstrated by several original experiments to be superior to the standard treatment in rebleeding prevention. Yet, early TIPS has gained wide application in recent years, as patients undergoing TIPS treatment have demonstrated clear benefits in reducing all-cause mortality and incidence of hepatic encephalopathy^5–7^. This may be related to the timing of TIPS implementation or severity of underlying liver disease. Specifically, cases in which TIPS was ineffective or inadequate included those of patients who died before receiving TIPS, patients who died soon after rescue TIPS, patients deemed too ill for TIPS due to liver failure, and patients in whom TIPS did not sufficiently reduce portal pressures^5,7^.

There is still some controversy regarding the application of early TIPS in cirrhotic portal hypertension. Clinicians still have some confusion when choosing this treatment, and there is an urgent need for more reliable evidentiary support from evidence-based medicine research. As a systematic review and meta-analysis, this study aims to comprehensively summarize the high-quality published research to provide more clear evidence-based medical evidence for clinical decision-making.

Early TIPS is TIPS performed within 72 h after diagnostic endoscopy^5^. With the recent introduction and application of early TIPS, current studies have shown its advantages over the standard treatment of all-cause mortality^5,7,8^. However, three network meta-analyses (NMAs)^9–11^ only explained the effects of endoscopic therapy, drug therapy, and surgery on mortality and adverse events and paid little attention to TIPS, either including only one article or not including any. Furthermore, there was no discussion on early TIPS.

This study aimed to compare the effects of early TIPS, non-early TIPS, and standard treatment in patients with cirrhosis and acute variceal bleeding. Owing to the paucity of direct comparison studies for some interventions, we used NMA to enable indirect comparisons across all treatments, allowing comprehensive utilization of all study data to assess the relative efficacy of the interventions, and it allows for direct comparisons between early TIPS and non-early TIPS, as well as indirect comparisons with the standard treatment group. By including studies where the control group received standard treatment, the NMA approach enables a larger sample size than traditional meta-analyses, providing a more robust analysis.

## Methods

### Protocol

The present NMA was conducted in accordance with the Preferred Reporting Items for Systematic Reviews and Meta-Analyses (PRISMA)^12^ and Assessing the Methodological Quality of Systematic Reviews (AMSTAR)^13^ Guidelines. The protocol of this review has been registered with the International Prospective Register of Systematic Reviews, but due to the double-blind peer review process, we are unable to provide the registration number. The NMA component of this study was designed and conducted by a statistical expert, an author with epidemiology and biostatistics degrees and extensive experience in NMA.

### Literature search

Through computer and manual search of the PubMed, Embase, Cochrane Library, ClinicalTrials.gov, and World Health Organization-approved trial registry databases, the search time was the establishment of the database to 31 October 2022; the search terms were “TIPS” and “transjugular intrahepatic portosystemic shunt”, among others. No language restrictions were applied. The detailed search strategy is shown in SDC, Supplementary Material.

### Study selection

#### Inclusion criteria

1. Patients: Patients with cirrhosis and acute variceal bleeding.
2. Intervention: Early TIPS, defined as early TIPS, which included all patients who underwent TIPS treatment within 72 h after the diagnostic endoscopy regardless of whether they received endoscopic sclerotherapy or any other form of hemostatic treatment during the initial endoscopic procedure.
3. Comparator: Non-early TIPS, refers to TIPS procedures performed beyond the 72-h timeframe following the diagnostic endoscopy. It includes patients who may have received endoscopic sclerotherapy or other hemostatic treatments during the initial endoscopic procedure but subsequently underwent TIPS treatment after the 72-h window. Standard treatment, defined as endoscopic techniques plus drug treatment or endoscopic techniques^7^.
4. Outcomes: The main outcomes were all-cause mortality and rebleeding. The secondary outcome was hepatic encephalopathy and new or worsening ascites.
5. Study designs: Randomized controlled trials (RCTs).

#### Exclusion criteria

1. Non-RCTs, cohort studies, crossover trials, case–control studies, retrospective studies, protocols, reviews, case reports, and ongoing trials lacking results.
2. Studies with an unidentified or unclear design or duplicate publications.
3. The time from bleeding to TIPS was not described.
4. Full text was unavailable.

### Data extraction and risk of bias assessment

The data extraction process was conducted by two independent reviewers who adhered to the eligibility criteria and reached a consensus in cases of discrepancy. The data extracted from all eligible studies, including the first author, publication year, country, sample size, age of participants, intervention type, Child–Pugh class, bleeding to TIPS time, and follow-up time, were systematically documented. In case of incomplete information, the corresponding author was contacted through e-mail to obtain any missing data. Any disagreements in the extracted data were resolved through discussion or, if necessary, adjudicated by a third reviewer.

Two reviewers independently used the Cochrane risk-of-bias tool 2 (RoB-2)^14^ to determine the individual bias for all included RCTs, which contained the following five items: bias related to the randomization process, deviations from intended interventions, missing outcome data, outcome measurement, and selection of the reported result. Each item was classified as ‘low risk’, ‘high risk’, or ‘some concerns’. To ensure the robustness of the risk of bias assessment, discrepancies were resolved through rigorous discussion between the reviewers or, if deemed necessary, by adjudication from a third reviewer.

### Certainty of evidence

We used the Confidence in Network Meta-Analysis (CINeMA) tool to assess the risk of bias within individual studies and evaluate the confidence in treatment comparisons in our NMA^15,16^. The CINeMA tool employs a framework that considers six key domains of bias or uncertainty: bias within studies, reporting bias, indirectness, imprecision, heterogeneity, and incoherence. We used the summary risk of bias rating to evaluate within-study bias. The confidence rating was classified as ‘high’ (low risk of bias), ‘moderate’ (moderate risk of bias), ‘low’ (high risk of bias), or ‘very low’ (very high risk of bias).

### Outcome definitions

The primary outcomes included all-cause mortality and rebleeding. The secondary outcomes were hepatic encephalopathy and new or worsening ascites.

All outcomes were binary, and the estimates are presented as odds ratio (OR) with 95% credible interval (CrI). Unlike traditional confidence intervals, which are based on frequentist statistical theory, CrIs are derived from Bayesian analysis theory. They incorporate both the information from the sample data and prior distribution information to express the uncertainty range of parameters. In this study, we chose to use CrIs because they excel at integrating multiple sources of information, including external data, and incorporating the researcher’s prior knowledge into parameter estimation, which traditional confidence intervals do not possess. We employed a non-informative uniform prior distribution in our CrI estimation, which means that no subjective prior knowledge was included, ensuring objectivity in our analysis^17^. Treatment success was defined as an OR and 95% CrI of <1. Following this, we calculated the relative ranking of therapies and ranked all therapies using surface under the cumulative ranking curve (SUCRA) value (range 0–100%), with higher values indicating a higher likelihood of an intervention ranking higher in efficacy.

### Data synthesis and analysis

#### Pairwise meta-analysis

The present study employed the ADDIS software to conduct random-effects model pairwise meta-analyses of all comparisons, encompassing standard treatment, for every outcome of interest. In addition, heterogeneity analysis was performed using the aforementioned software. Specifically, an *I*^2^ value of <40% was considered indicative of the low heterogeneity, whereas a higher *I*^2^ value was considered indicative of heterogeneity^14^.

#### NMA

Network plots were created using StataSE 15.0 (64-bit), which presents the connection between different interventions.

R software was used to perform heterogeneity analysis^14^. Heterogeneity was assessed based on *I*^2^; an *I*^2^ value of <40% was considered indicative of low heterogeneity, whereas a higher *I*^2^ value was considered indicative of heterogeneity^14^.

NMA was performed using GeMTC software package, which is a Bayesian NMA package. The primary analysis was conducted using a random-effects model, which allows for heterogeneity across studies and provides estimates of treatment effects in a more conservative manner. Node-splitting tests were initially employed to assess the consistency and transitivity of effect estimates. In cases where closed loops could not be formed, rendering the consistency analysis infeasible, a visual examination of direct and indirect effect estimates was conducted to identify any notable discrepancies^18^. The analyses were performed with four Markov chains, and a potential scale reduction factor (PSRF) analysis was subsequently performed to generate results^19^.

### Subgroup and sensitivity analyses

In the subgroup analysis, we evaluated the impact of study period on outcomes by dividing the included RCTs into two subgroups based on the year of study publication, using 2007 as the cutoff point. This cutoff was chosen as it was the median publication year for all included RCTs. The two subgroups were pre-2007 RCTs before 2007 and post-2007 RCTs after 2007. This subgroup analysis was performed to assess whether improvements in techniques and experience over time influenced outcomes based on the publication year of trials. Sensitivity analyses were conducted using a change analysis model (fixed and random models). The implementation of sensitivity analyses was premeditated or undertaken upon the request of the reviewer or adherence to established protocol.

### Publication bias

StataSE 15.0 (64-bit) was used to analyze potential publication bias across the included studies through the evaluation of comparison-adjusted funnel plots. Symmetric distribution of data points (representing individual studies) on both sides of the null line in a funnel plot indicates the absence of publication bias and small study effects^14^.

## Results

### Study selection

**Figure 1:**
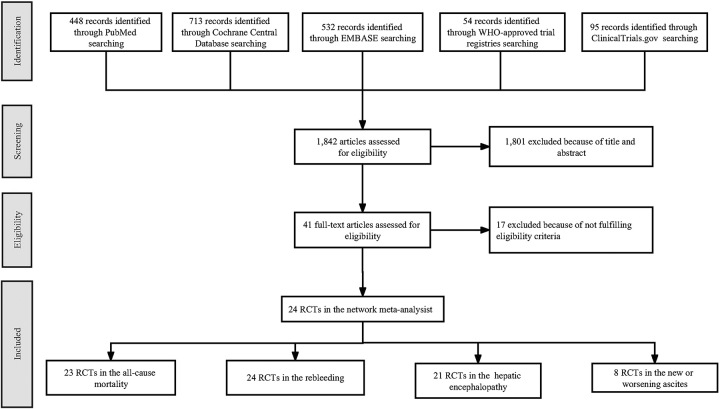
PRISMA process. PRISMA, Preferred Reporting Items for Systematic Reviews and Meta-Analyses.

In the preliminary search phase, a comprehensive search of relevant databases yielded a total of 1842 citations. Subsequently, an automated duplicate removal process was implemented, which resulted in the exclusion of 627 duplicate records. Next, 1174 articles were discarded through a meticulous screening process based on their title and abstract. Following this, a thorough assessment of the full texts of the remaining articles was conducted, leading to the exclusion of 17 articles. Among these, two articles were deemed ineligible because of the inability to retrieve the original manuscript or establish communication with the corresponding author to procure the necessary data. Consequently, a total of 24 RCTs^5,7,8,20–40^ fulfilled the prespecified inclusion criteria and were subsequently incorporated into the NMA. The selection process for these studies is depicted in the PRISMA process (Fig. 1), providing a comprehensive visualization of the methodology employed for study inclusion.

### Study characteristics

Twenty-four articles with 1894 participants were included in the study. Nine articles compared early TIPS with standard treatment, whereas 15 compared non-early TIPS with standard treatment. The duration of follow-up in the included studies was 1–2.63 years. The basic characteristics of the included studies are listed in SDC Table 1 (Supplemental Digital Content 1, http://links.lww.com/JS9/B279).

### Quality assessment

The RoB-2 findings for 24 eligible RCTs are shown in SDC Figure 1 (Supplemental Digital Content 1, http://links.lww.com/JS9/B279). All included studies were deemed to have a ‘low risk’ of bias regarding the random sequence generation. This was attributed to the detailed account provided by each study regarding their methods of randomization, such as employing a random number table, balanced block randomization, or software-generated randomized number. Moreover, all studies were appraised as having a ‘low risk’ of bias concerning the ‘randomization process’ as they explicitly delineated their allocation approach, which involved using sealed envelopes.

### All-cause mortality

**Figure 2:**
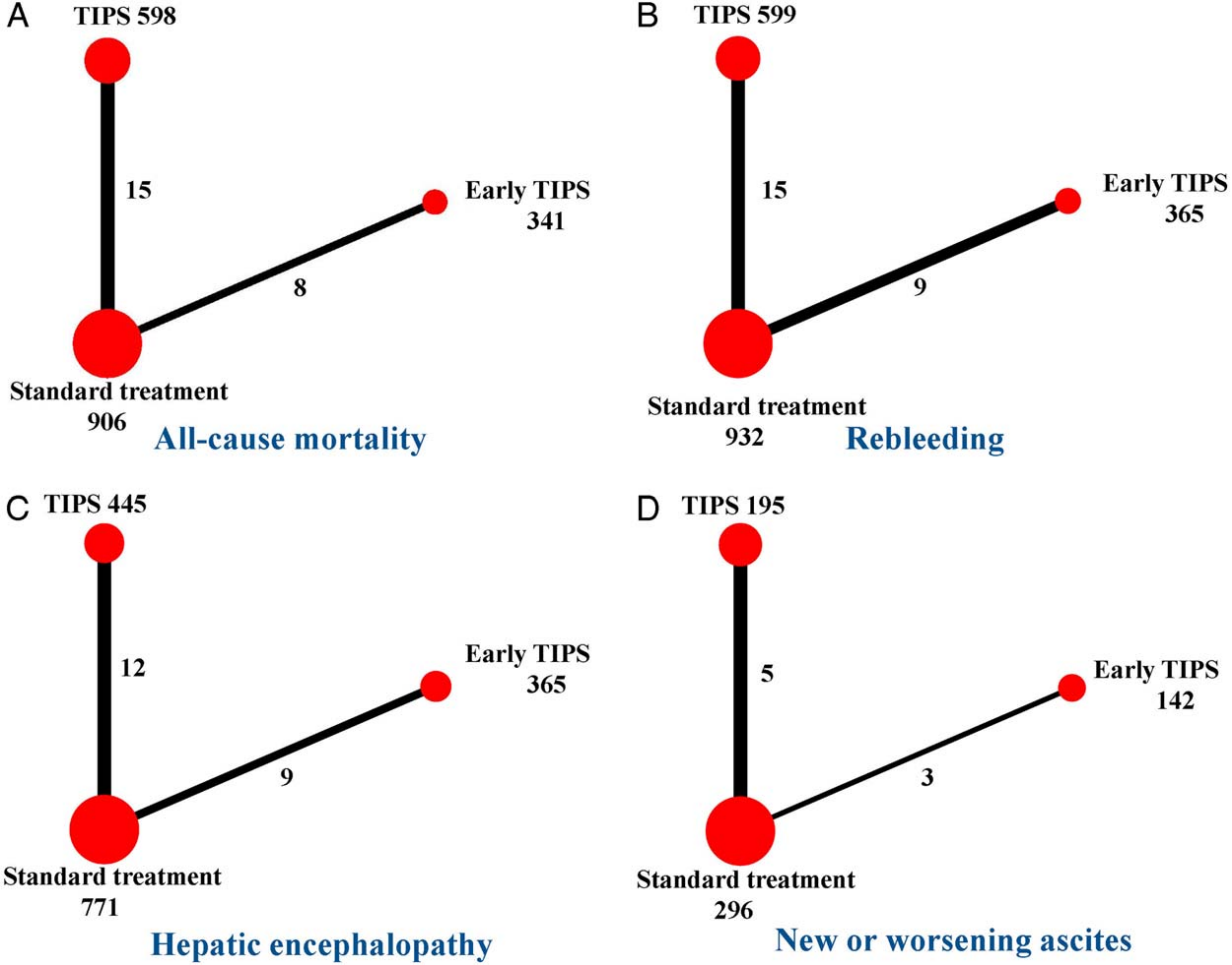
Network plots correlating TIPS and standard treatment for patients with cirrhosis and acute variceal bleeding. Each circle represents an intervention, called node. Node size is related to the number of patients receiving an intervention. The line between nodes represents a direct comparison, and its thickness is proportional to the number of tests for each comparison. TIPS, transjugular intrahepatic portosystemic shunt.

**Figure 3:**
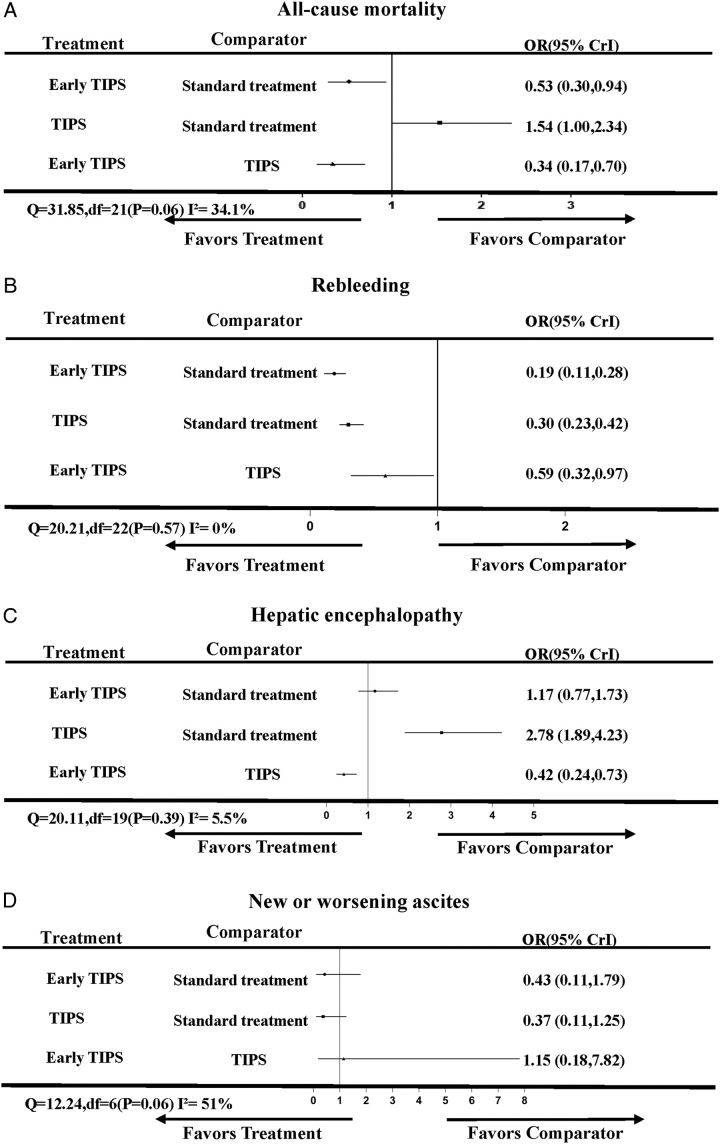
Forest plot of each treatment to the comparator. The plot shows the OR value and 95% CrI between each treatment to comparator; CrI, credible interval; OR, odds ratio; TIPS, transjugular intrahepatic portosystemic shunt.

**Figure 4:**
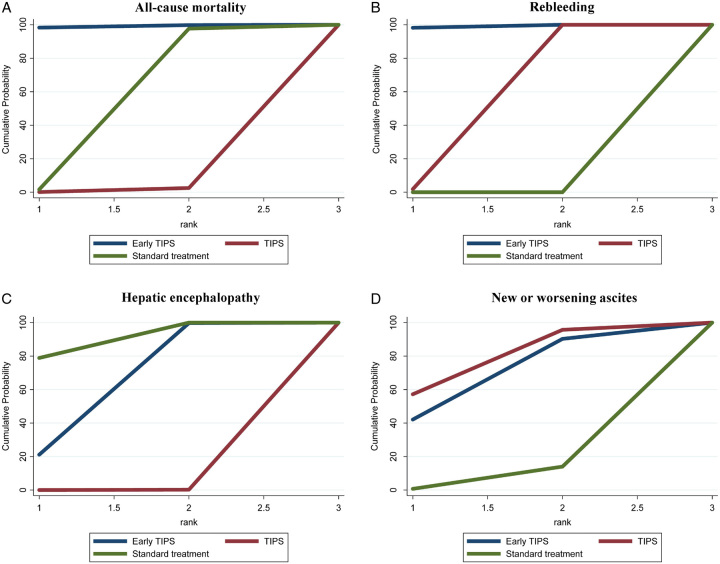
SUCRA (surface under the cumulative ranking curve) value graph. The curves represent interventions. The larger the area under the curve, the higher the likelihood that the intervention is the optimal choice. TIPS, transjugular intrahepatic portosystemic shunt.

Twenty-three articles^5,7,8,20,21,23–40^ reported all-cause mortality. Since there were no direct comparisons between early TIPS and non-early TIPS, the follow-up durations were as follows: For studies comparing early-TIPS versus standard treatment, the median (25th–75th percentile) follow-up was 1.7 years (1.1–2.0 years). For studies comparing non-early TIPS versus standard treatment, the median (25th–75th percentile) follow-up was 2.0 years (1.3–2.5 years). In the network plot, three nodes represent early TIPS, non-early TIPS, or standard treatment. The three line-linked nodes represent direct comparison, which were 8 for the early TIPS to standard treatment and 15 for the non-early TIPS to standard treatment (Fig. 2A). Upon conducting 50 000 simulations, the PSRF, an indicator of model convergence, was determined to be 1.00. Compared with standard treatment, early TIPS (OR, 0.53; 95% CrI, 0.30–0.94; SUCRA, 98.3; Figs 3A, 4A) reduced all-cause mortality (moderate-to-high-quality evidence; SDC Fig. 2, Supplemental Digital Content 1, http://links.lww.com/JS9/B279). The results showed low heterogeneity (*I*^2^=34.1%; Fig. 3A). Moreover, given the inability to form loop connections, the assessment of consistency could not be operationalized, but the results from the consistency model and the inconsistency model showed negligible differences in the obtained ORs and confidence intervals. This implies an arguably high level of consistency across studies (SDC Table 2, Supplemental Digital Content 1, http://links.lww.com/JS9/B279). We also found consistent results between the pairwise and NMAs (SDC Table 3, Supplemental Digital Content 1, http://links.lww.com/JS9/B279). The funnel plot indicated no publication bias (SDC Figure 6, Supplemental Digital Content 1, http://links.lww.com/JS9/B279).

### Rebleeding

Twenty-four articles^5,7,8,20–40^ reported rebleeding. The three nodes represent early TIPS, non-early TIPS, or standard treatment. The three line-linked nodes represent direct comparison, which were 9 for the early TIPS to standard treatment and 15 for the non-early TIPS to standard treatment (Fig. 2B). Upon conducting 50 000 simulations, the PSRF was determined to be 1.00. Compared with standard treatment, early TIPS (OR, 0.19; 95% CrI, 0.11–0.28; SUCRA, 98.2; Figs 3B, 4B) and non-early TIPS (OR, 0.30; 95% CrI, 0.23–0.42; SUCRA, 1.8; Figs 3B, 4B) reduced the risk of rebleeding (moderate-to-high-quality evidence; SDC Fig. 3, Supplemental Digital Content 1, http://links.lww.com/JS9/B279). Low heterogeneity was observed (*I*^2^=0%; Fig. 4B). Moreover, given the inability to form loop connections, the assessment of consistency could not be operationalized, but the results from the consistency model and the inconsistency model showed negligible differences in the obtained ORs and confidence intervals. This implies an arguably high level of consistency across studies (SDC Table 2, Supplemental Digital Content 1, http://links.lww.com/JS9/B279). We found consistent results between the pairwise and NMAs (SDC Table 3, Supplemental Digital Content 1, http://links.lww.com/JS9/B279). There was no publication bias (SDC Figure 7, Supplemental Digital Content 1, http://links.lww.com/JS9/B279).

### Hepatic encephalopathy

Twenty-one articles^5,7,8,22–36,38–40^ compared three nodes representing early TIPS, non-early TIPS, and standard treatment. The three line-linked nodes represent direct comparison, which were 9 for the early TIPS to standard treatment and 12 for the non-early TIPS to standard treatment (Fig. 2C). Upon conducting 50 000 simulations, the PSRF was determined to be 1.00. Compared with standard treatment, early TIPS was not associated with a reduced risk of hepatic encephalopathy. Non-early TIPS (OR, 2.78; 95% CrI, 1.89–4.23; SUCRA, 0; Figs 3C, 4C) could induce or exacerbate hepatic encephalopathy (moderate-to-high-quality evidence; SDC Fig. 4, Supplemental Digital Content 1, http://links.lww.com/JS9/B279). However, low heterogeneity was observed (*I*^2^=5.5%; Fig. 3C). Moreover, given the inability to form loop connections, the assessment of consistency could not be operationalized, but the results from the consistency model and the inconsistency model showed negligible differences in the obtained ORs and confidence intervals. This implies an arguably high level of consistency across studies (SDC Table 2, Supplemental Digital Content 1, http://links.lww.com/JS9/B279). The findings of the pairwise meta-analysis conformed to the NMA results (SDC Table 3, Supplemental Digital Content 1, http://links.lww.com/JS9/B279). The funnel plot indicated no publication bias (SDC Fig. 8, Supplemental Digital Content 1, http://links.lww.com/JS9/B279).

### New or worsening ascites

Eight articles^5,7,25,30,34,38–40^ compared three nodes representing early TIPS, non-early TIPS, and standard treatment. The three line-linked nodes represent direct comparison, which were 5 for the early TIPS to standard treatment and 3 for the non-early TIPS to standard treatment (Fig. 2D). Upon conducting 50 000 simulations, the PSRF was determined to be 1.00. No treatment was superior to the standard treatment (moderate-to-high-quality evidence; SDC Figure 5, Supplemental Digital Content 1, http://links.lww.com/JS9/B279), although early TIPS was associated with a lower OR value (0.43; SUCRA, 42.1%; Figs 3D, 4D). Moderate heterogeneity was observed (*I*^2^=51%; Fig. 3D). The funnel plot indicated no publication bias (SDC Figure 9, Supplemental Digital Content 1, http://links.lww.com/JS9/B279).

### Subgroup and sensitivity analyses

In the subgroup analysis, we divided the studies into two groups, pre-2007 and post-2007, using the median publication year of all RCTs as the cutoff point. Subsequently, we conducted subgroup analyses for the primary outcome measures. For all-cause mortality, our results indicated no statistically significant differences between the pre-2007 and post-2007 groups when compared after standard treatment. However, when the results were pooled, early TIPS was superior to standard treatment. In the case of hepatic encephalopathy, early TIPS showed an advantage over standard treatment in the pre-2007 subgroup analysis, but the pooled results did not show a statistically significant difference. Overall, we did not find a significant effect of the publication year of trials on efficacy. Sensitivity analysis results aligned with the main results (SDC Tables 4, 5, Supplemental Digital Content 1, http://links.lww.com/JS9/B279).

## Discussion

On the basis of moderate-to-high-quality evidence, we concluded that early TIPS is associated with reduced all-cause mortality and rebleeding. Although early TIPS and standard treatment had a comparable incidence of hepatic encephalopathy, early TIPS was superior to non-early TIPS in this regard. Some recent studies have also reported that TIPS-related hepatic encephalopathy can be controlled^41,42^. In addition, one study found that early TIPS placement improved survival at admission in patients at high risk for hepatic encephalopathy, suggesting that hemorrhage may be a precipitating factor for hepatic encephalopathy and that early TIPS placement helps control bleeding and thus address a precipitating factor for hepatic encephalopathy rapidly^43^. To date, there have been no trials comparing early TIPS and non-early TIPS. Hence, this study, to our best knowledge, is the first, and the results show that early TIPS is superior, not only to standard treatment, but also to non-early TIPS.

Considering technological advancements, we hypothesized that later trials might yield slightly better results compared with earlier trials. However, our subgroup analysis did not observe this trend, possibly due to the small sample size. Overall, our study did not find the publication year of the trials significantly affected efficacy. Nevertheless, this potential effect is worth further evaluation in future studies with larger sample sizes.

Acute variceal bleeding frequently complicates cirrhosis and carries a high mortality risk, urgently requiring prompt and effective control methods that can significantly reduce mortality and complications^44,45^. For acute variceal bleeding, the current standard treatment remains a combination of vasoactive agents, prophylactic antibiotics, endoscopic therapy, and TIPS, which is often used as a salvage treatment or second-line therapy, but several multicenter studies have shown that only 7–10% of patients had early TIPS placement^46,47^.

Recently, the application of early TIPS has become a frequently discussed topic, and multiple RCTs have indicated that early TIPS reduces the risk of rebleeding from acute variceal bleeding in patients with cirrhosis compared with standard treatment. However, other studies have reported conflicting results regarding the effect of early TIPS and standard treatment on mortality and hepatic encephalopathy^7,8,40^. Moreover, three studies showed that early TIPS significantly improved survival in patients with cirrhosis and acute variceal bleeding compared to standard treatment. Significant survival benefits are particularly evident in high-risk patients (Child–Pugh B and Child–Pugh C) with active bleeding during endoscopy^48–50^, which is consistent with the conclusion of this study. However, both studies included fewer than five RCTs and included a large number of observational studies. Our study further found that early TIPS did not increase the risk of hepatic encephalopathy compared with standard treatment. A study published in 2022 indicated that early TIPS may not reduce mortality at 6 months, which contradicts our findings. It is worth noting that the meta-analysis published in 2022 included a limited number of studies, with only four RCTs out of nine included. The small sample size might explain the discrepancy in the results^51^. Regarding NMA, a recent study published in Cochrane^11^ showed that compared with endoscopic treatment, TIPS has advantages in improving rebleeding but may increase the risk of other complications, which contradicts the conclusion of our study. However, that study included very few RCTs in which the intervention was TIPS, and there were no RCTs on early TIPS.

However, there are many clinical factors that should be considered when selecting TIPS: non-malignant portal vein thrombosis, refractory ascites, recurrent symptomatic hepatic hydrothorax, failed treatment of gastroesophageal varices with nonselective beta-blockers (NSBBs) and variceal ligation, Child class C with score <14 or Child class B with active bleeding, bridge treatment in patients listed for liver transplantation, transfusion-dependent portal hypertensive gastropathy cases in which NSBBs fail or are not tolerated, treatment of gastroesophageal varices type 2 or isolated varices type 1 with TIPS placement with or without embolization, hepatorenal syndrome type 2 associated with refractory/recidivant ascites, failure of anticoagulation and mechanical revascularization in Budd–Chiari syndrome cases with persistent ascites or other related complications, and patients with porto-sinusoidal vascular disorder^52,53^.

Before performing a TIPS procedure, it is essential to consider whether the patient exhibits the following factors: very advanced liver disease (Child–Pugh score >13 points), recurrent episodes of overt hepatic encephalopathy without an identifiable precipitating factor, heart failure, pulmonary hypertension, hepatic vein obstruction, or anatomical alterations due to prior surgical procedures^54^.

Nonetheless, our study has some limitations. First, this study employed NMA to compare treatment effects, but this approach comes with inherent limitations related to the combination of diverse comparisons, such as heterogeneity arising from different studies that may affect the results. Additionally, the inclusion of a limited number of studies also restricts the applicability of the method. Second, it is difficult to quantify specific factors, such as inconsistencies in the measures of standard treatment across different eras and studies, such as variations in endoscopic hemostasis methods, and treatment intervals; these factors may introduce potential heterogeneity and affect the results, and they lead to unknown bias. Third, some studies with small sample sizes resulted in low power. Finally, the included clinical trials in this study exhibited variability in follow-up times, preventing us from conducting stratified analyses for different standardized follow-up time points. This could potentially affect the assessment of long-term efficacy. Future research should routinely report clinical outcomes at standard follow-up times to achieve more reliable comparisons of long-term efficacy.

## Conclusion

Based on the moderate-to-high quality evidence presented in this study, early TIPS placement was associated with reduced all-cause mortality [with a median follow-up of 1.9 years (25th–75th percentile range 1.9–2.3 years)] and rebleeding compared to standard treatment and non-early TIPS. Although early TIPS and standard treatment had a comparable incidence of hepatic encephalopathy, early TIPS showed superiority over non-early TIPS in this aspect. Recent studies have also shown promising results in controlling TIPS-related hepatic encephalopathy. However, it is important to consider individual patient characteristics and weigh the potential benefits against the risks associated with early TIPS. Therefore, we recommend that clinicians carefully evaluate the patient’s condition, considering factors such as the severity of variceal bleeding, underlying liver disease, and overall clinical status, before making a treatment decision. Further, well-designed RCTs comparing early TIPS with non-early TIPS are needed to validate these findings and provide more definitive guidance.

## Ethical approval

Not applicable.

## Consent

Not applicable.

## Sources of funding

This work was supported by the Young Scientists Fund of the Natural Science Foundation of Hunan Province (Grant No. 2021JJ40964).

## Author contribution

Y.H. and X.Y.: designed the study; X.W. and Y.H.: conducted the data analysis; X.W., X.L., S.S., Y.X., and X.Y.: contributed to the writing and revision of the manuscript.

## Conflicts of interest disclosure

There are no conflicts of interest to declare.

## Research registration unique identifying number (UIN)

1. Name of the registry: PROSPERO.
2. Unique identifying number or registration ID: CRD42022377965.
3. Hyperlink to your specific registration (must be publicly accessible and will be checked): https://www.crd.york.ac.uk/PROSPERO/display_record.php?RecordID=377965.

## Guarantor

Xinbo Yin.

## Data availability statement

The data that supports the findings of this study are available in the supplementary material of this article.

## Provenance and peer review

Not commissioned, externally peer-reviewed.

## Supplementary Material

## References

[1] Wilson R Williams DM . Cirrhosis. Med Clin North Am 2022;106:437–446. 35491064 10.1016/j.mcna.2021.12.001

[2] Ginès P Krag A Abraldes JG . Liver cirrhosis. Lancet 2021;398:1359–1376. 34543610 10.1016/S0140-6736(21)01374-X

[3] Jakab SS Garcia-Tsao G . Evaluation and management of esophageal and gastric varices in patients with cirrhosis. Clin Liver Dis 2020;24:335–350. 32620275 10.1016/j.cld.2020.04.011 PMC11090175

[4] Molleston JP Bennett WE Jr . Mortality, risk factors and disparities associated with esophageal variceal bleeding in children’s hospitals in the US. J Pediatr 2021;232:176–182. 33450222 10.1016/j.jpeds.2020.12.082

[5] García-Pagán JC Caca K Bureau C . Early use of TIPS in patients with cirrhosis and variceal bleeding. New Engl J Med 2010;362:2370–2379. 20573925 10.1056/NEJMoa0910102

[6] de Franchis R . Expanding consensus in portal hypertension: Report of the Baveno VI Consensus Workshop: Stratifying risk and individualizing care for portal hypertension. J Hepatol 2015;63:743–752. 26047908 10.1016/j.jhep.2015.05.022

[7] Lv Y Yang Z Liu L . Early TIPS with covered stents versus standard treatment for acute variceal bleeding in patients with advanced cirrhosis: a randomised controlled trial. Lancet Gastroenterol Hepatol 2019;4:587–598. 31153882 10.1016/S2468-1253(19)30090-1

[8] Dunne P Sinha R Stanley AJ . Randomised clinical trial: standard of care versus early-transjugular intrahepatic porto-systemic shunt (TIPSS) in patients with cirrhosis and oesophageal variceal bleeding. Aliment Pharmacol Ther 2020;52:98–106. 32452561 10.1111/apt.15797

[9] Roberts D Best LM Freeman SC . Treatment for bleeding oesophageal varices in people with decompensated liver cirrhosis: a network meta-analysis. Cochrane Database Syst Rev 2021;4:Cd013155. 33837526 10.1002/14651858.CD013155.pub2 PMC8094233

[10] Roccarina D Best LM Freeman SC . Primary prevention of variceal bleeding in people with oesophageal varices due to liver cirrhosis: a network meta-analysis. Cochrane Database Syst Rev 2021;4:Cd013121. 33822357 10.1002/14651858.CD013121.pub2 PMC8092414

[11] Plaz Torres MC Best LM Freeman SC . Secondary prevention of variceal bleeding in adults with previous oesophageal variceal bleeding due to decompensated liver cirrhosis: a network meta-analysis. Cochrane Database Syst Rev 2021;3:Cd013122. 33784794 10.1002/14651858.CD013122.pub2 PMC8094621

[12] Page MJ McKenzie JE Bossuyt PM . The PRISMA 2020 statement: An updated guideline for reporting systematic reviews. Int J Surg 2021;88:105906. 33789826 10.1016/j.ijsu.2021.105906

[13] Shea BJ Reeves BC Wells G . AMSTAR 2: a critical appraisal tool for systematic reviews that include randomised or non-randomised studies of healthcare interventions, or both. BMJ 2017;358:j4008. 28935701 10.1136/bmj.j4008 PMC5833365

[14] Higgins JPT Thomas J Chandler J , Cochrane Handbook for Systematic Reviews of Interventions, 2019. 10.1002/14651858.ED000142 PMC10284251 31643080

[15] Nikolakopoulou A Higgins JPT Papakonstantinou T . CINeMA: an approach for assessing confidence in the results of a network meta-analysis. PLoS Med 2020;17:e1003082. 32243458 10.1371/journal.pmed.1003082 PMC7122720

[16] Papakonstantinou T Nikolakopoulou A Higgins JPT . CINeMA: Software for Semiautomated Assessment of the Confidence in the Results of Network Meta-analysis. Wiley; 2020. 10.1002/cl2.1080 PMC8356302 37131978

[17] Kelter R . The evidence interval and the Bayesian evidence value: on a unified theory for Bayesian hypothesis testing and interval estimation. Br J Math Stat Psychol 2022;75:550–592. 36200811 10.1111/bmsp.12267

[18] Dias S Welton NJ Caldwell DM . Checking consistency in mixed treatment comparison meta-analysis. Stat Med 2010;29:932–944. 20213715 10.1002/sim.3767

[19] Brooks SP Gelman A . General methods for monitoring convergence of iterative simulations. J Comput Graph Stat 1998;7:434–455.

[20] des Anastomoses Intra Groupe d’Etude. TIPS vs sclerotherapy+ propranolol in the prevention of variceal rebleeding: preliminary results of a multicenter randomized trial. Hepatology 1995;22:A297.

[21] Cabrera J Maynar M Granados R . Transjugular intrahepatic portosystemic shunt versus sclerotherapy in the elective treatment of variceal hemorrhage. Gastroenterology 1996;110:832–839. 8608893 10.1053/gast.1996.v110.pm8608893

[22] Cello JP Ring EJ Olcott EW . Endoscopic sclerotherapy compared with percutaneous transjugular intrahepatic portosystemic shunt after initial sclerotherapy in patients with acute variceal hemorrhage: a randomized, controlled trial. Ann Intern Med 1997;126:858–865. 9163286 10.7326/0003-4819-126-11-199706010-00002

[23] Jalan R Finlayson ND Hayes PC . A randomized trial comparing transjugular intrahepatic portosystemic stent-shunt with variceal band ligation in the prevention of rebleeding from esophageal varices. Hepatology 1997;26:1115–1122. 9362350 10.1002/hep.510260505

[24] Rössle M Deibert P Haag K . Randomised trial of transjugular-intrahepatic-portosystemic shunt versus endoscopy plus propranolol for prevention of variceal rebleeding. Lancet 1997;349:1043–1049. 9107241 10.1016/s0140-6736(96)08189-5

[25] Sanyal AJ Freedman AM Luketic VA . Transjugular intrahepatic portosystemic shunts compared with endoscopic sclerotherapy for the prevention of recurrent variceal hemorrhage: a randomized, controlled trial. Ann Intern Med 1997;126:849–857. 9163285 10.7326/0003-4819-126-11-199706010-00001

[26] Sauer P Theilmann L Stremmel W . Transjugular intrahepatic portosystemic stent shunt versus sclerotherapy plus propranolol for variceal rebleeding. Gastroenterology 1997;113:1623–1631. 9352865 10.1053/gast.1997.v113.pm9352865

[27] Merli M Salerno F Riggio O . Transjugular intrahepatic portosystemic shunt versus endoscopic sclerotherapy for the prevention of variceal bleeding in cirrhosis: a randomized multicenter trial. Hepatology 1998;27:48–53. 9425916 10.1002/hep.510270109

[28] Sauer P Benz C Theilmann L . Transjugular intrahepatic portosystemic stent shunt (TIPS) vs. endoscopic banding in the prevention of variceal rebleeding: final results of a randomized study. Gastroenterology 1998;114:A1334.

[29] García-Villarreal L Martínez-Lagares F Sierra A . Transjugular intrahepatic portosystemic shunt versus endoscopic sclerotherapy for the prevention of variceal rebleeding after recent variceal hemorrhage. Hepatology 1999;29:27–32. 9862845 10.1002/hep.510290125

[30] Narahara Y Kanazawa H Kawamata H . A randomized clinical trial comparing transjugular intrahepatic portosystemic shunt with endoscopic sclerotherapy in the long-term management of patients with cirrhosis after recent variceal hemorrhage. Hepatol Res 2001;21:189–198. 11673103 10.1016/s1386-6346(01)00104-8

[31] Pomier-Layrargues G Villeneuve JP Deschênes M . Transjugular intrahepatic portosystemic shunt (TIPS) versus endoscopic variceal ligation in the prevention of variceal rebleeding in patients with cirrhosis: a randomised trial. Gut 2001;48:390–396. 11171831 10.1136/gut.48.3.390 PMC1760139

[32] Gülberg V Schepke M Geigenberger G . Transjugular intrahepatic portosystemic shunting is not superior to endoscopic variceal band ligation for prevention of variceal rebleeding in cirrhotic patients: a randomized, controlled trial. Scand J Gastroenterol 2002;37:338–343. 11916197 10.1080/003655202317284255

[33] Sauer P Hansmann J Richter GM . Endoscopic variceal ligation plus propranolol vs. transjugular intrahepatic portosystemic stent shunt: a long-term randomized trial. Endoscopy 2002;34:690–697. 12195325 10.1055/s-2002-33565

[34] Monescillo A Martínez-Lagares F Ruiz-del-Arbol L . Influence of portal hypertension and its early decompression by TIPS placement on the outcome of variceal bleeding. Hepatology 2004;40:793–801. 15382120 10.1002/hep.20386

[35] Lo G-H Liang HL Chen WC . A prospective, randomized controlled trial of transjugular intrahepatic portosystemic shunt versus cyanoacrylate injection in the prevention of gastric variceal rebleeding. Endoscopy 2007;39:679–685. 17661241 10.1055/s-2007-966591

[36] Luo X Wang Z Tsauo J . Advanced cirrhosis combined with portal vein thrombosis: a randomized trial of TIPS versus endoscopic band ligation plus propranolol for the prevention of recurrent esophageal variceal bleeding. Radiology 2015;276:286–293. 25759969 10.1148/radiol.15141252

[37] Sauerbruch T Mengel M Dollinger M . Prevention of rebleeding from esophageal varices in patients with cirrhosis receiving small-diameter stents versus hemodynamically controlled medical therapy. Gastroenterology 2015;149:660–8.e1. 25989386 10.1053/j.gastro.2015.05.011

[38] Holster IL Tjwa ETTL Moelker A . Covered transjugular intrahepatic portosystemic shunt versus endoscopic therapy+ β-blocker for prevention of variceal rebleeding. Hepatology 2016;63:581–589. 26517576 10.1002/hep.28318

[39] Lv Y Qi X He C . Covered TIPS versus endoscopic band ligation plus propranolol for the prevention of variceal rebleeding in cirrhotic patients with portal vein thrombosis: a randomised controlled trial. Gut 2018;67:2156–2168. 28970291 10.1136/gutjnl-2017-314634

[40] Chen Y Ma X Zhang X . Prevention of variceal rebleeding in cirrhotic patients with advanced hepatocellular carcinoma receiving molecularly targeted therapy: a randomized pilot study transjugular intrahepatic portosystemic shunt versus endoscopic plus β-blocker. Hepatol Int 2022;16:1379–1389. 36255564 10.1007/s12072-022-10388-7

[41] Madoff DC Wallace MJ Ahrar K . TIPS-related hepatic encephalopathy: management options with novel endovascular techniques. Radiographics 2004;24:21–36; discussion 36-7. 14730033 10.1148/rg.241035028

[42] Pereira K Carrion AF Martin P . Current diagnosis and management of post-transjugular intrahepatic portosystemic shunt refractory hepatic encephalopathy. Liver Int 2015;35:2487–2494. 26332169 10.1111/liv.12956

[43] Rudler M Hernández-Gea V Procopet BD . Hepatic encephalopathy is not a contraindication to pre-emptive TIPS in high-risk patients with cirrhosis with variceal bleeding. Gut 2023;72:749–758. 36328772 10.1136/gutjnl-2022-326975

[44] Ardevol A Ibañez-Sanz G Profitos J . Survival of patients with cirrhosis and acute peptic ulcer bleeding compared with variceal bleeding using current first-line therapies. Hepatology 2018;67:1458–1471. 28714072 10.1002/hep.29370

[45] Thabut D Rudler M Lebrec D . Early TIPS with covered stents in high-risk patients with cirrhosis presenting with variceal bleeding: are we ready to dive into the deep end of the pool? J Hepatol 2011;55:1148–1149. 21708107 10.1016/j.jhep.2011.05.013

[46] Angeli P Bernardi M Villanueva C . EASL Clinical Practice Guidelines for the management of patients with decompensated cirrhosis. J Hepatol 2018;69:406–460. 29653741 10.1016/j.jhep.2018.03.024

[47] Garcia‐Tsao G Abraldes JG Berzigotti A . Portal hypertensive bleeding in cirrhosis: risk stratification, diagnosis, and management: 2016 practice guidance by the American Association for the study of liver diseases. Hepatology 2017;65 ). 310–335. 27786365 10.1002/hep.28906

[48] Zhou GP Jiang YZ Sun LY . Early transjugular intrahepatic portosystemic shunt for acute variceal bleeding: a systematic review and meta-analysis. Eur Radiol 2021;31:5390–5399. 33409783 10.1007/s00330-020-07525-x

[49] Nicoară-Farcău O Han G Rudler M . Effects of early placement of transjugular portosystemic shunts in patients with high-risk acute variceal bleeding: a meta-analysis of individual patient data. Gastroenterology 2021;160:193–205.e10. 32980344 10.1053/j.gastro.2020.09.026

[50] Li S Zhang C Lin LL . Early-TIPS versus current standard therapy for acute variceal bleeding in cirrhosis patients: a systemic review with meta-analysis. Front Pharmacol 2020;11 603. 10.3389/fphar.2020.00603 PMC7282546 32581776

[51] Hussain I Wong YJ Lohan R . Does preemptive transjugular intrahepatic portosystemic shunt improve survival after acute variceal bleeding? Systematic review, meta-analysis, and trial sequential analysis of randomized trials. J Gastroenterol Hepatol 2022;37:455–463. 34665473 10.1111/jgh.15714

[52] Lapenna L Di Cola S Gazda J . New indications for TIPSs: What do we know so far? J Clin Exp Hepatol 2023;13:794–803. 37693277 10.1016/j.jceh.2023.01.017 PMC10483008

[53] Mammen S Keshava SN Kattiparambil S . Acute portal vein thrombosis, no longer a contraindication for transjugular intrahepatic porto-systemic shunt (TIPS) insertion. J Clin Exp Hepatol 2015;5:259–261. 26628844 10.1016/j.jceh.2014.08.008 PMC4632077

[54] García-Pagán JC Saffo S Mandorfer M . Where does TIPS fit in the management of patients with cirrhosis? JHEP Rep 2020;2:100122. 32671331 10.1016/j.jhepr.2020.100122 PMC7347999

