# Supplementary material for: Comparative efficacy of early TIPS, Non-early TIPS, and Standard treatment in patients with cirrhosis and acute variceal bleeding: a network meta-analysis
Source: Int J Surg. 2023 Nov 3;110(2):1149–58. doi: 10.1097/JS9.0000000000000865 (PMC10871647; doi:10.1097/JS9.0000000000000865)
Supplement: Supplementary file 1 [file js9-110-1149-s001.pdf]

Supplementary Material

SDC, Search strategy

Pubmed

(("esophageal and gastric varices"[MeSH Terms] OR (("oesophageal"[Title/Abstract] OR "esophageal"[Title/Abstract]) AND ("varic*"[Title/Abstract] OR "varix*"[Title/Abstract]))) AND ("portasystemic shunt, transjugular intrahepatic"[MeSH Terms] OR "portasystemic shunt, surgical"[MeSH Terms] OR (("transjugular"[Title/Abstract] OR "portosystemic"[Title/Abstract] OR "portasystemic"[Title/Abstract] OR "portacaval"[Title/Abstract] OR "mesocaval"[Title/Abstract] OR "splenorenal"[Title/Abstract] OR "surgical"[Title/Abstract] OR "radiological"[Title/Abstract] OR "intrahepatic"[Title/Abstract] OR "selective"[Title/Abstract] OR "non-selective"[Title/Abstract] OR "partial"[Title/Abstract] OR "total"[Title/Abstract]) AND ("shunt*"[Title/Abstract] OR "anastomos*"[Title/Abstract])) OR ("h shunt*"[Title/Abstract] OR "TIPS"[Title/Abstract] OR "PSS"[Title/Abstract]) OR (("varicose veins"[MeSH Terms] OR ("varicos*"[Title/Abstract] AND ("vein*"[Title/Abstract] OR "veno*"[Title/Abstract])) OR ("tortu*"[Title/Abstract] AND ("vein*"[Title/Abstract] OR "veno*"[Title/Abstract])) OR ("incomp*"[Title/Abstract] AND ("vein*"[Title/Abstract] OR "veno*"[Title/Abstract] OR "valv*"[Title/Abstract])) OR ("insuffic*"[Title/Abstract] AND ("vein*"[Title/Abstract] OR "veno*"[Title/Abstract]))) AND ("ligation"[MeSH Terms] OR ("ligation*"[Title/Abstract] OR "banding*"[Title/Abstract]))) OR ("sclerotherapy"[MeSH Terms] OR "sclerosing solutions"[MeSH Terms] OR "sclero*"[Title/Abstract]))) AND (randomizedcontrolledtrial[Filter])

Embase

(esophageal AND 'gastric varices'/exp OR ((oesophageal:ti,ab,kw OR esophageal:ti,ab,kw) AND (varic*:ti,ab,kw OR varix*:ti,ab,kw))) AND ('portasystemic shunt transjugular intrahepatic'/exp OR 'portasystemic shunt, surgical'/exp OR ((transjugular:ti,ab,kw OR portosystemic:ti,ab,kw OR portasystemic:ti,ab,kw OR portacaval:ti,ab,kw OR mesocaval:ti,ab,kw OR splenorenal:ti,ab,kw OR surgical:ti,ab,kw OR radiological:ti,ab,kw OR intrahepatic:ti,ab,kw OR selective:ti,ab,kw OR non-selective:ti,ab,kw OR partial:ti,ab,kw OR total:ti,ab,kw) AND (shunt*:ti,ab,kw OR anastomos*:ti,ab,kw)) OR h-shunt*:ti,ab,kw OR tips:ti,ab,kw OR pss:ti,ab,kw OR (('varicose vein'/exp OR (varicos*:ti,ab,kw AND (vein*:ti,ab,kw OR veno*:ti,ab,kw)) OR (tortu*:ti,ab,kw AND (vein*:ti,ab,kw OR veno*:ti,ab,kw)) OR (incomp*:ti,ab,kw AND (vein*:ti,ab,kw OR veno*:ti,ab,kw OR valv*:ti,ab,kw)) OR (insuffic*:ti,ab,kw AND (vein*:ti,ab,kw OR veno*:ti,ab,kw))) AND ('ligation'/exp OR ligation*:ti,ab,kw OR banding*:ti,ab,kw)) OR 'sclerotherapy'/exp OR 'sclerosing solutions'/exp OR sclero*:ti,ab,kw) AND (((random*:ti,ab,kw OR factorial*:ti,ab,kw OR crossover*:ti,ab,kw OR 'cross over*':ti,ab,kw OR cross-over*:ti,ab,kw OR placebo*:ti,ab,kw OR double*:ti,ab,kw) AND 'adj blind*':ti,ab,kw OR single*:ti,ab,kw) AND 'adj blind*':ti,ab,kw OR assign*:ti,ab,kw OR allocat*:ti,ab,kw OR volunteer*:ti,ab,kw OR 'randomized controlled trial'/exp OR 'crossover-procedure' OR 'single-blind procedure')

Cochrane Library

#1 (Esophageal and Gastric Varices) or((((oesophageal or esophageal) and (varic* or varix*))):ti,ab,kw)

#2 MeSH descriptor: [Peritoneovenous Shunt] explode all trees

#3 ((portasystemic shunt transjugular intrahepatic):ti,ab,kw) OR (((transjugular or portosystemic or portasystemic or portacaval or mesocaval or splenorenal or surgical or radiological or intrahepatic or

selective or non-selective or partial or total) and (shunt* or anastomos*)):ti,ab,kw) OR ((H-shunt* or TIPS or PSS):ti,ab,kw)

#4 #2 or #3

#5 MeSH descriptor: [Varicose Veins] explode all trees

#6 (((varicos* and (vein* or veno*))):ti,ab,kw) or (((tortu* and (vein* or veno*))):ti,ab,kw) or (((incomp* and (vein* or veno* or valv*))):ti,ab,kw) or (((insuffic* and (vein* or veno*))):ti,ab,kw)

#7 MeSH descriptor: [Ligation] explode all trees

#8 (ligation* or banding*):ti,ab,kw

#9 (#5 or #6) and (#7 or #8)

#10 MeSH descriptor: [Sclerotherapy] explode all trees

#11 MeSH descriptor: [Sclerosing Solutions] explode all trees

#12 (sclero*):ti,ab,kw

#13 #10 or #11 or #12

#14 #1 and (#4 or #9 or #13)

#15 ((Randomized Controlled Trial):ti,ab,kw) or ((controlled clinical trial):ti,ab,kw) or ((Randomized or placebo or drug therapy or randomly or trial or groups):ti,ab,kw)

#16 #14 and #15

SDC, Figure 1. Risk of bias 2 summary

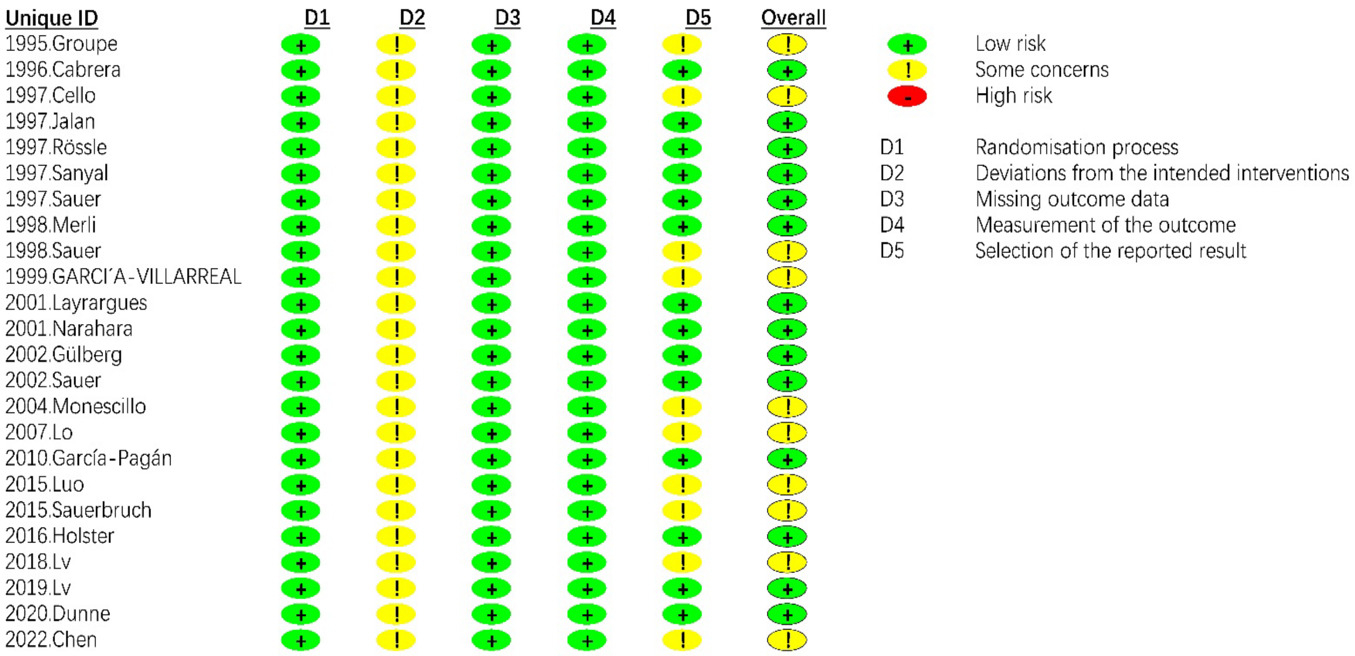

SDC, Figure 2. Within-study bias assessment for all-cause mortality

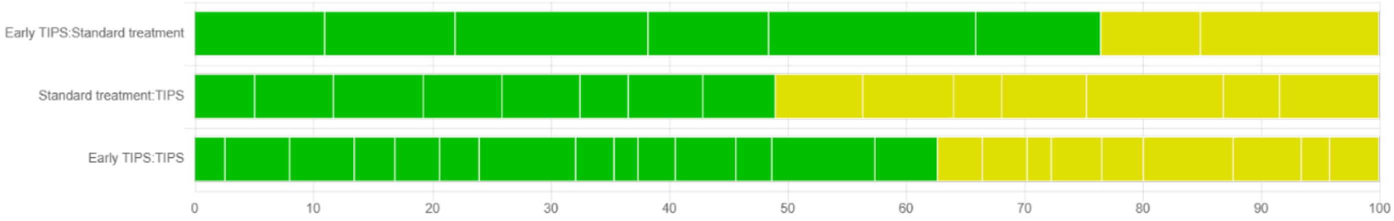

Note. The y-axis displays each comparison. The x-axis represents the percentage of studies with respective low risks of bias (green) versus unclear risks of bias (yellow). TIPS, transjugular intrahepatic portosystemic shunt.

SDC, Figure 3. Within-study bias assessment for rebleeding

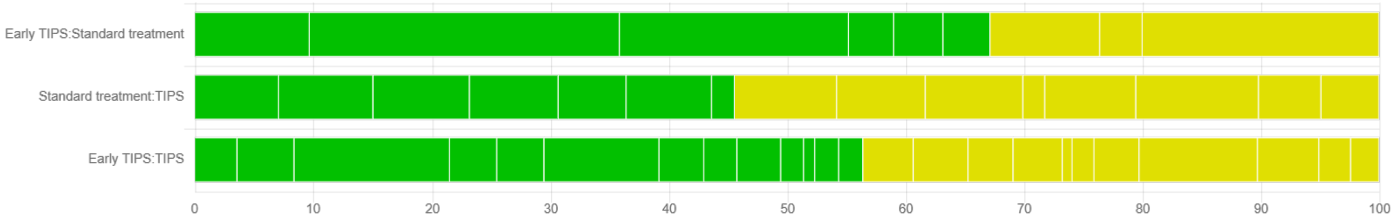

Note. The y-axis displays each comparison. The x-axis represents the percentage of studies with respective

low risks of bias (green) versus unclear risks of bias (yellow). TIPS, transjugular intrahepatic portosystemic shunt.

SDC, Figure 4. Within-study bias assessment for hepatic encephalopathy

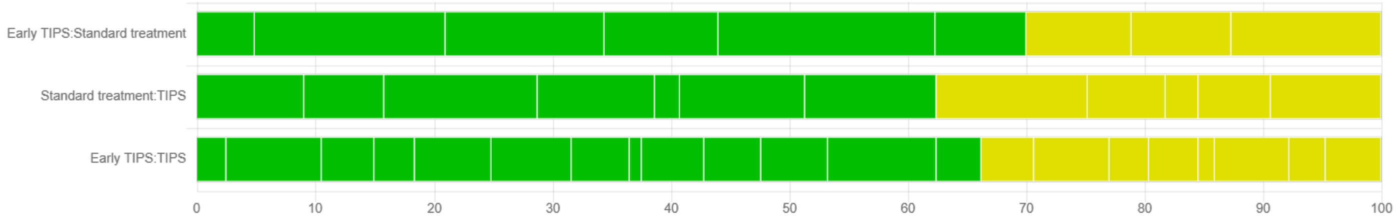

Note. The y-axis displays each comparison. The x-axis represents the percentage of studies with respective low risks of bias (green) versus unclear risks of bias (yellow). TIPS, transjugular intrahepatic portosystemic shunt.

SDC, Figure 5. Within-study bias assessment for new or worsening ascites

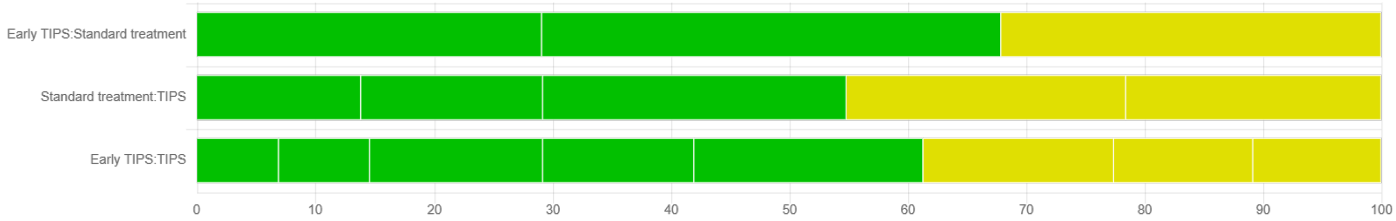

Note. The y-axis displays each comparison. The x-axis represents the percentage of studies with respective low risks of bias (green) versus unclear risks of bias (yellow). TIPS, transjugular intrahepatic portosystemic shunt.

SDC, Figure 6. Funnel plot of all-cause mortality

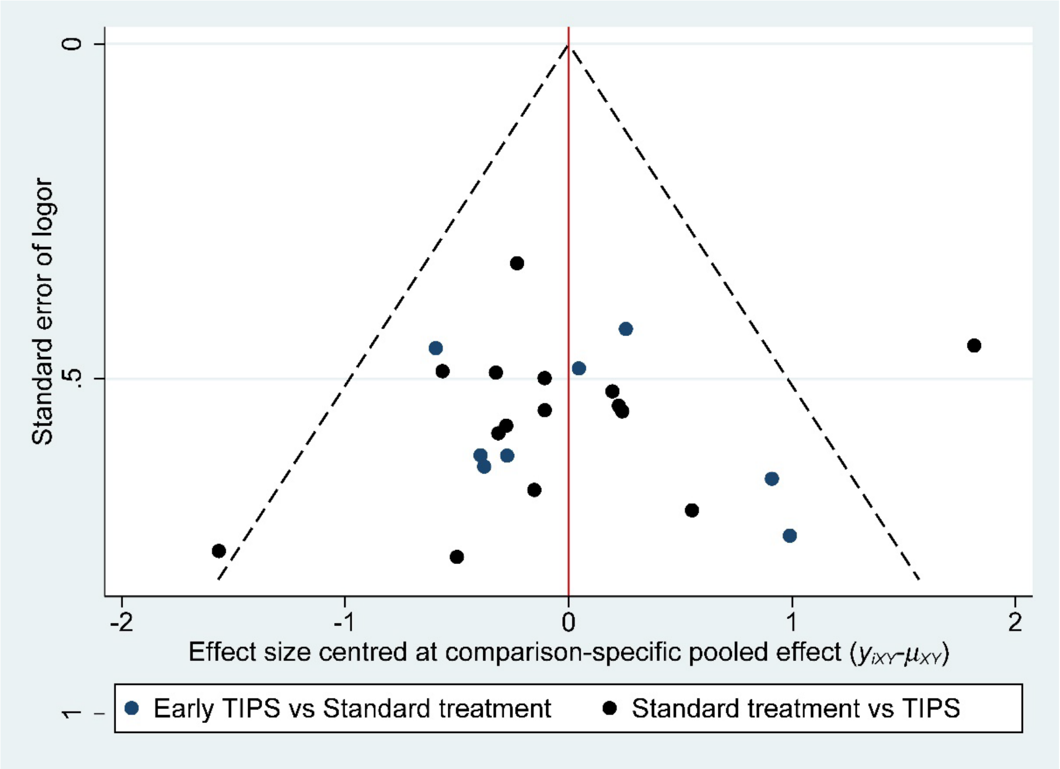

Note: TIPS, transjugular intrahepatic portosystemic shunt.

SDC, Figure 7. Funnel plot of rebleeding

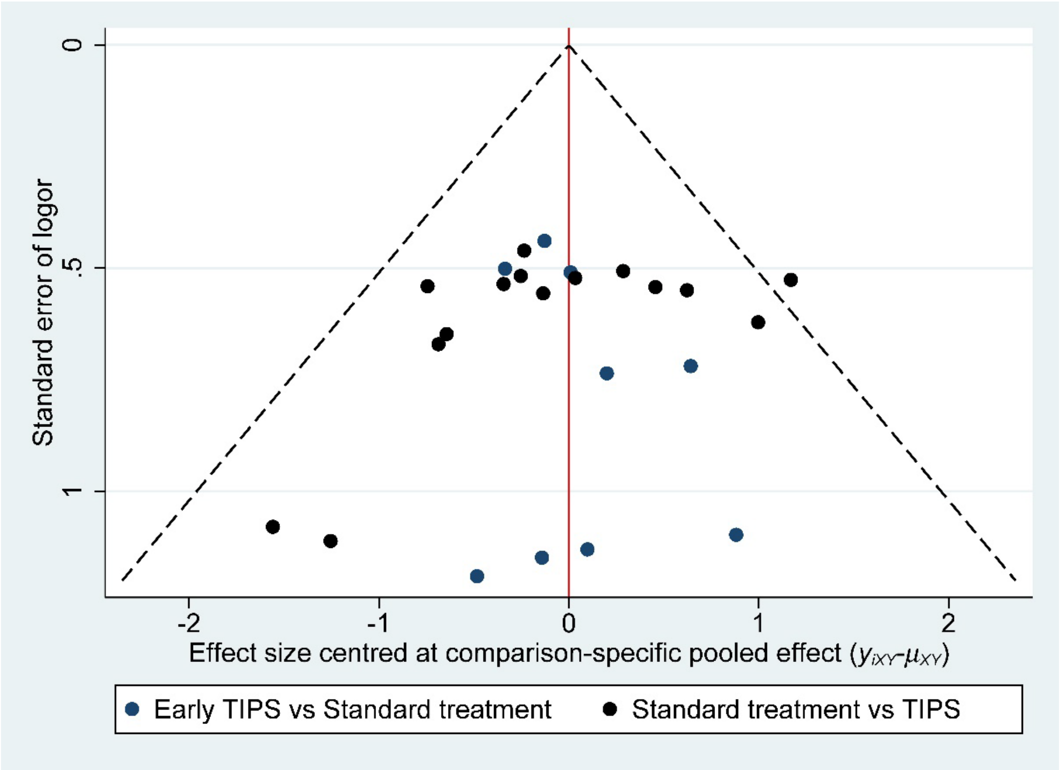

Note: TIPS, transjugular intrahepatic portosystemic shunt.

SDC, Figure 8. Funnel plot of hepatic encephalopathy

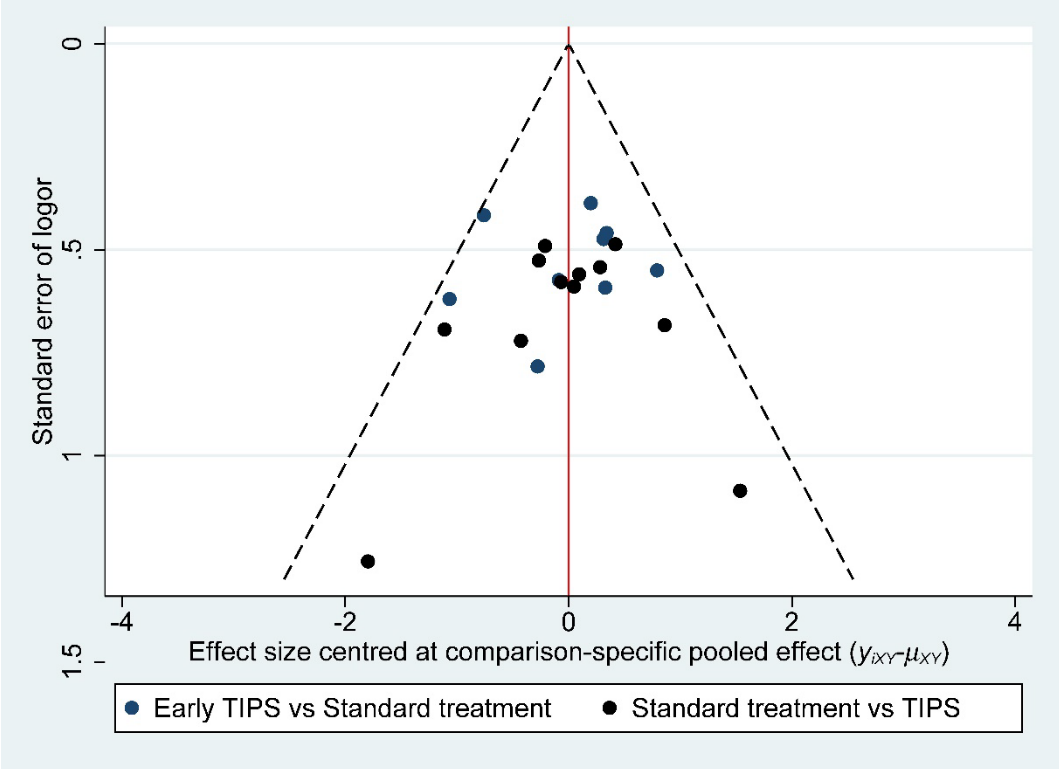

Note: TIPS, transjugular intrahepatic portosystemic shunt.

SDC, Figure 9. Funnel plot of new or worsening ascites

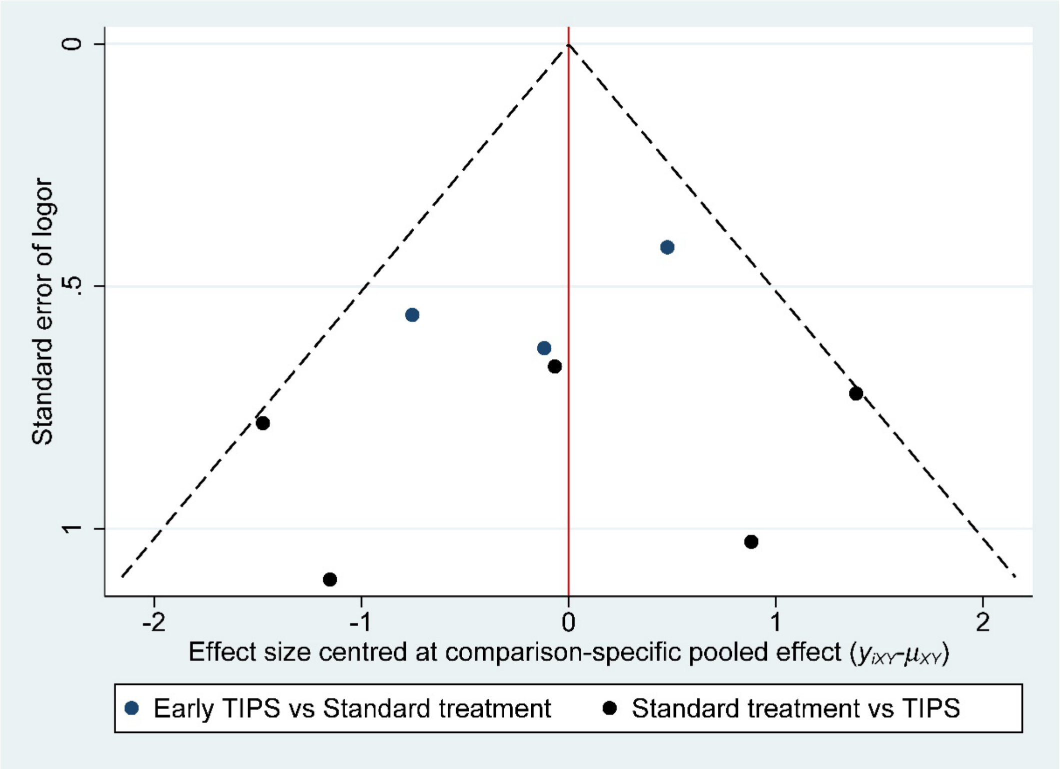

Note: TIPS, transjugular intrahepatic portosystemic shunt.

SDC, Table 1. Characteristics of the included studies

Study ID	Country	Study size	Age (years, mean±SD)	Sex (male/female)	Treatment	Child-Pugh class			Diagnostic endoscopy to TIPS time (hours)	Follow-up time (years)
						A	B	C		
1995.Groupe	France	65	51	46/19	Standard treatment (33)<br>TIPS (32)	0	0	65	>72	1
1996.Cabrera	Spain	63	56±11	43/20	Standard treatment (32)<br>TIPS (31)	28	29	6	>72	1.24
1997.Cello	USA	49	48±2	36/13	Early TIPS (24)<br>Standard treatment (25)	N/A	N/A	N/A	48	1.57
1997.Jalan	UK	58	57±9	37/21	Early TIPS (31)<br>Standard treatment (27)	7	23	28	24	1.38
1997.Rössle	Germany	126	56±12	84/42	Early TIPS (61)<br>Standard treatment (65)	39	64	23	48	1.15
1997.Sanyal	USA	80	50±7	53/26	Standard treatment (39)<br>TIPS (41)	13	28	39	>72	2.61
1997.Sauer	Germany	83	57±12	35/48	Standard treatment (41)<br>TIPS (42)	27	36	20	>72	2
1998.Merli	Italy	81	59±10	58/23	Standard treatment (43)<br>TIPS (38)	26	45	10	>72	1.46
1998.Sauer	Germany	85	N/A	N/A	Standard treatment (27)<br>TIPS (43)	N/A	N/A	N/A	>72	1.35
1999.GARCI´A-VILLARREAL	Spain	46	57±9	37/9	Standard treatment (24)<br>TIPS (22)	8	24	14	>72	2.08
2001.Layrargues	Canada	80	54±12	56/24	Early TIPS (41)<br>Standard treatment (39)	N/A	N/A	N/A	57	1.86

2001.Narahara	Japan	78	53±2	62/16	Standard treatment (40)<br>TIPS (38)	N/A	N/A	N/A	>72	2.59
2002.Gülberg	Germany	54	57±2	39/15	Standard treatment (26)<br>TIPS (28)	21	27	6	>72	2
2002.Sauer	Germany	85	54±12	50/25	Standard treatment (42)<br>TIPS (43)	25	35	25	>72	1
2004.Monescillo	Spain	52	58±12	41/11	Early TIPS (26)<br>Standard treatment (26)	7	21	24	24	1
2007.Lo	China	72	54±8	53/19	Standard treatment (37)<br>TIPS (35)	21	39	12	>72	2.63
2010.García-Pagán	European	63	51±8	44/19	Early TIPS (32)<br>Standard treatment (31)	0	32	31	<72	2
2015.Luo	China	73	50±14	43/30	Early TIPS (37)<br>Standard treatment (36)	0	49	24	<72	1.87
2015.Sauerbruch	Germany	185	55±10	125/60	Standard treatment (95)<br>TIPS (90)	93	N/A	N/A	>72	2.48
2016.Holster	Netherland	72	55±11	41/31	Standard treatment (35)<br>TIPS (37)	26	37	9	>72	1.89
2018.Lv	China	49	48±5	29/20	Early TIPS (24)<br>Standard treatment (25)	19	27	3	>72	2
2019.Lv	China	129	51±11	87/42	Early TIPS (84)<br>Standard treatment (45)	0	100	29	<72	2
2020.Dunne	UK	58	51±11	39/19	Early TIPS (29)<br>Standard treatment (29)	0	25	33	<72	1
2022.Chen	China	108	55±2	88/18	Standard treatment (54)<br>TIPS (54)	7	73	26	>72	2.4

Note. SD, standard deviation; TIPS, transjugular intrahepatic portosystemic shunt; N/A, not applicable; UK, United Kingdom; USA, United States of America.

SDC, Table 2. Outcomes of network meta-analysis based on the consistency model versus the inconsistency model

**All-cause mortality (Consistency model)**		
Early TIPS	1.88 (1.06, 3.35)	2.91 (1.42, 5.79)
0.53 (0.30, 0.94)	Standard care	1.54 (1.00, 2.34)
0.34 (0.17, 0.70)	0.65 (0.43, 1.00)	TIPS
**All-cause mortality (Inconsistency model)**		
Early TIPS	1.88 (1.05, 3.42)	2.88 (1.43, 5.97)
0.53 (0.29, 0.95)	Standard care	1.54 (1.00, 2.33)
0.35 (0.17, 0.70)	0.65 (0.43, 1.00)	TIPS
**Rebleeding (Consistency model)**		
Early TIPS	5.22 (3.62, 9.20)	1.71 (1.03, 3.11)
0.19 (0.11, 0.28)	Standard care	0.30 (0.23, 0.42)
0.59 (0.32, 0.97)	3.29 (2.39, 4.32)	TIPS
**Rebleeding (Inconsistency model)**		
Early TIPS	5.70 (3.60, 9.42)	1.77 (1.02, 3.17)
0.18 (0.11, 0.28)	Standard care	0.31 (0.22, 0.43)

0.57 (0.32, 0.98)	3.23 (2.34, 4.49)	TIPS
**Hepatic encephalopathy (Consistency model)**		
Early TIPS	0.86 (0.58, 1.29)	2.39 (1.37, 4.17)
1.17 (0.77, 1.73)	Standard care	2.78 (1.89, 4.23)
0.42 (0.24, 0.73)	0.36 (0.24, 0.53)	TIPS
**Hepatic encephalopathy (Inconsistency model)**		
Early TIPS	0.83 (0.57, 1.28)	2.33 (1.34, 4.30)
1.20 (0.78, 1.76)	Standard care	2.81 (1.93, 4.23)
0.43 (0.23, 0.75)	0.36 (0.24, 0.52)	TIPS
**New or worsening ascites (Consistency model)**		
Early TIPS	2.33 (0.56, 9.40)	0.87 (0.13, 5.65)
0.43 (0.11, 1.79)	Standard care	0.37 (0.11, 1.25)
1.15 (0.18, 7.82)	2.70 (0.80, 8.88)	TIPS
**New or worsening ascites (Inconsistency model)**		
Early TIPS	2.32 (0.55, 9.37)	0.86 (0.13, 5.11)
0.43 (0.11, 1.80)	Standard care	0.37 (0.11, 1.22)
1.16 (0.20, 7.93)	2.70 (0.82, 9.24)	TIPS

Note: TIPS, transjugular intrahepatic portosystemic shunt.

SDC, Table 3. Pairwise MA and NMA consistency/inconsistency model of early TIPS and TIPS with standard treatment as the reference

Intervention	Pairwise MA OR (95% CI)	NMA consistency model OR (95% CrI)	NMA inconsistency model OR (95% CrI)	Confidence rating
**All-cause mortality**				
Early TIPS	0.56 (0.38, 0.82)	0.53 (0.30, 0.94)	0.53 (0.29,0.95)	High
TIPS	0.66 (0.44, 1.00)	0.65 (0.43, 1.00)	0.65 (0.43, 1.00)	High
**Rebleeding**				
Early TIPS	0.19 (0.12, 0.29)	0.19 (0.11, 0.28)	0.18 (0.11, 0.28)	High
TIPS	0.33 (0.24, 0.46)	0.30 (0.23, 0.42)	0.31 (0.22, 0.43)	High
**Hepatic encephalopathy**				
Early TIPS	1.17 (0.80, 1.70)	1.17 (0.77, 1.73)	1.20 (0.78, 1.76)	High
TIPS	2.68 (1.90, 3.79)	2.78 (1.89, 4.23)	2.81 (1.93, 4.23)	High
**New or worsening ascites**				
Early TIPS	0.43 (0.20, 0.90)	0.43 (0.11, 1.79)	0.43 (011, 1.80)	High
TIPS	0.42 (0.14, 1.26)	0.37 (0.11, 1.25)	0.37 (0.11, 1.22)	High

Note. MA, meta-analysis; NMA, network meta-analysis; OR, odds ratio; CI, confidence interval; CrI, credible interval; TIPS, transjugular intrahepatic

portosystemic shunt.

SDC, Table 4. Subgroup-analysis of early TIPS and TIPS with standard treatment

Group	Early TIPS	TIPS
**All-cause mortality**		
**Pre-2007 (1995–2007)**	0.64 (0.35, 1.25)	1.20 (0.76, 1.81)
**Post-2007 (2008–2022)**	0.45 (0.12, 1.65)	2.75 (0.78, 9.96)
**Overall (1995–2022)**	0.53 (0.30, 0.94)	1.54 (1.00, 2.34)
**Rebleeding**		
**Pre-2007 (1995–2007)**	0.17 (0.09, 0.32)	0.37 (0.25, 0.54)
**Post-2007 (2008–2022)**	0.18 (0.06, 0.48)	0.17 (0.06, 0.37)
**Overall (1995–2022)**	0.19 (0.11, 0.28)	0.30 (0.23, 0.42)
**Hepatic encephalopathy**		
**Pre-2007 (1995–2007)**	0.41 (0.21, 0.88)	3.23 (1.84, 5.00)
**Post-2007 (2008–2022)**	0.48 (0.15, 1.60)	2.17 (0.86, 5.48)
**Overall (1995–2022)**	0.24 (0.24, 0.73)	2.78 (1.89, 4.23)
**New or worsening ascites**		
**Pre-2007 (1995–2007)**	0.82 (0.08, 7.73)	0.33 (0.04, 2.26)
**Post-2007 (2008–2022)**	0.32 (0.05, 2.25)	0.38 (0.08, 1.93)
**Overall (1995–2022)**	0.43 (0.11, 1.79)	0.37 (0.11, 1.25)

Note. TIPS, transjugular intrahepatic portosystemic shunt.

SDC, Table 5. Sensitivity-analysis of early TIPS and TIPS with standard treatment

Statistical model	Early TIPS	TIPS
**All-cause mortality**		
**Fixed model**	0.56 (0.38, 0.82)	1.51 (1.15, 1.97)
**Random model**	0.53 (0.30, 0.94)	1.54 (1.00, 2.34)
**Rebleeding**		
**Fixed model**	0.19 (0.12, 0.29)	0.33 (0.25, 0.45)
**Random model**	0.19 (0.11, 0.28)	0.30 (0.23, 0.42)
**Hepatic encephalopathy**		
**Fixed model**	1.17 (0.84, 1.62)	2.68 (1.90, 3.79)
**Random model**	1.17 (0.77, 1.73)	2.78 (1.89, 4.23)
**New or worsening ascites**		
**Fixed model**	0.44 (0.21, 0.89)	0.40 (0.22, 0.72)
**Random model**	0.43 (0.11, 1.79)	0.37 (0.11, 1.25)

Note. TIPS, transjugular intrahepatic portosystemic shunt.
